# Role of Autophagy in Cardiovascular Disease and Aging

**DOI:** 10.7759/cureus.20042

**Published:** 2021-11-30

**Authors:** Christos Koutouroushis, Oiendrila Sarkar

**Affiliations:** 1 General Internal Medicine, St. Mary's Hospital, Isle of Wight NHS Trust, Newport, GBR; 2 General Internal Medicine, St. Mary’s Hospital, Isle of Wight NHS Trust, Newport, GBR

**Keywords:** risk factors of cardiovascular diseases, mtor, sirtuins, igf-1 signalling, ampk, cellular aging, therapeutic fasting, caloric-restriction, autophagy

## Abstract

Cardiovascular disease is the leading cause of death worldwide and is expected to further increase as people continue to live even longer. Although the life span of the general population is increasing, the con of such a prolonged life span is that aging has certain detrimental effects on the molecular, structural, and functional elements of the cardiovascular system. This review will discuss various molecular pathways linked to longevity, most notably autophagy and its associated mechanisms, and how these pathways can be targeted to promote cardiovascular health through the process of aging. It is to be noted that the process of autophagy decreases with aging; hence, this review concludes that the promotion of autophagy, through implementation of caloric restriction, intermittent fasting, and pharmacologic agents, has proven to be an efficacious means of stimulating cardiovascular health. Therefore, autophagy is an important target for prevention and procrastination of cardiovascular pathologies in the geriatric population.

## Introduction and background

Aging and the incidence of cardiovascular disease

The population is aging [1]. Through the advent of technology, improved medicine, and zeal for better care facilities, humans are living longer. More people are likely to be employed, have better houses to live in, and have more money to spend on luxury. This is not necessarily a good thing. Even if people are living longer, a significant proportion of this increased life span can be spent in poor health. The root of this issue is largely due to the development of numerous chronic conditions, such as cardiovascular conditions, diabetes, strokes, joint disorders, cancers, and many others that emerge in greater prevalence with increased age [2]. These conditions can limit patients’ physical and mental capabilities, thus restricting their daily activities. This not only affects the geriatric population but also places a burden on their relatives and the health care system in terms of the support they will require.

This review focuses on aging and cardiovascular diseases. According to the World Health Organization, cardiovascular disease is the leading cause of death worldwide, with ~17.9 million people dying every year from such conditions [3]. Furthermore, in 2004, the UK government spent ~£29.1 billion to tackle cardiovascular ailments [4]. Hence, cardiovascular disease is not only the leading cause of morbidity worldwide but is also a massive financial burden on the world economy. As such, there is an increased need to develop preventative measures against age-related cardiovascular diseases.

## Review

Autophagy

**Figure 1:**
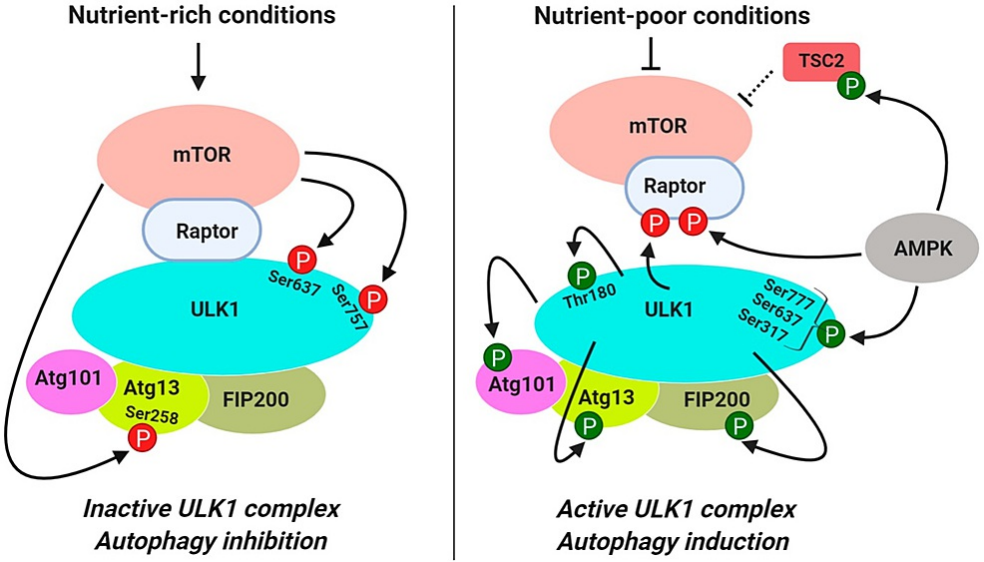
Autophagy regulation in response to nutrient cell status MTOR, Molecular target of rapamycin. Source: This figure was taken from Dossou et al. 2019 [13].

As stated by the National Institutes of Health, the definition of autophagy is a process by which a cell breaks down and destroys old, damaged, or abnormal proteins and other substances in its cytoplasm. The breakdown products are then recycled for important cell functions, especially during periods of stress or starvation [5]. In mammals (humans particularly), there are three main ways by which autophagy works to recycle the worn-out and damaged cells and cellular components in the body. The three processes of autophagy in mammals are (1) micro-autophagy, (2) chaperon-mediated autophagy, and (3) macro-autophagy [6]. Although the molecular machinery involved differs somewhat between the three processes, they share similar features. This includes how lysosomes are used during the process to degrade organelles and cytoplasmic proteins. (1) During micro-autophagy, the lysosomes themselves identify the proteins or organelles that need degradation and recycling. They attach to the membranes of the identified organelles and engulf them for degradation and recycling. (2) In chaperon-mediated autophagy, chaperons are used to identify proteins (pentapeptide-targeting motif) on the surface of organelles. These organelles are then transported by chaperons to lysosomes for degradation. (3) Macro-autophagy involves the de novo synthesis of cytosolic organelles, termed autophagosomes, which transfer worn-out and damaged organelles and proteins to lysosomes for degradation and recycling [7]. Several studies have suggested that autophagosomes (cytosolic vesicles) originate from the endoplasmic reticulum [8,9], but this process is not yet clear. Before their formation, autophagosomes are termed phagophores and are made up of a double membrane. Although the origins of the latter are not clearly understood, double membranes are thought to originate from the plasma membrane, endoplasmic reticulum, Golgi complex, and/or mitochondria [8-12]. The initiation of macro-autophagy, and subsequently the formation of phagophores, is considered to be regulated by the formation of a stable complex that is not affected by the nutrient status of the cell, which in turn is activated or deactivated by association with the molecular target of rapamycin complex 1 (MTORC1). This stable complex is composed of the Atg1 homolog (either ULK1 or ULK2), mammalian homolog Atg13, and the RB1-inducible coiled-coil [1], as depicted in Figure 1.

**Figure 2:**
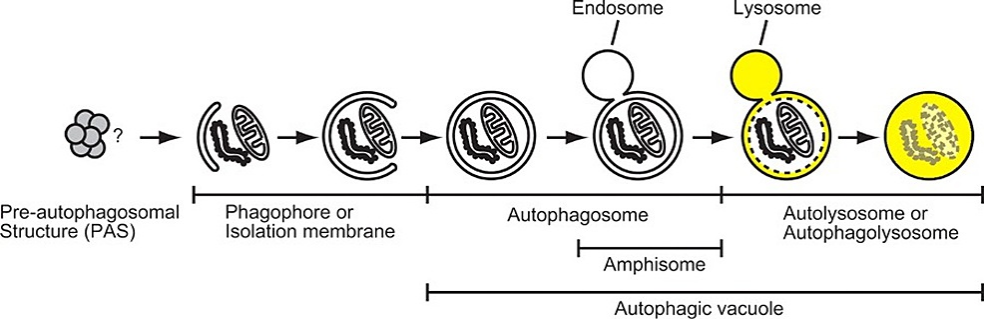
The steps involved in the formation of autophagosomes and lysosomal degradation of their contents Source: This figure was taken from Mizushima et al. 2007 [19].

When cells are starved of nutrients, or treated with rapamycin, molecular target of rapamycin complex 1 (MTORC1) dissociates from the complex, resulting in dephosphorylation, thus initiating macro-autophagy. As depicted in Figure 2, once the membrane of the phagophores is fully curved and the openings fuse, it is termed the autophagosome. The autophagosome moves along the microtubule network of the cellular cytoplasm to engulf the organelles targeted by autophagy. Once the organelles are engulfed, the autophagosome travels to fuse with lysosomes, a process mediated by the fusion of their outer membranes. The product of this fusion is termed the autolysosome. The association of the ultraviolet-resistance-associated gene (UVRAG) with the phosphatidylinositol 3-kinase (PtdIns3k) complex activates the GTPase ras-related protein Rab7 (GTPase RAB7), which promotes the fusion of lysosomes with autophagosomes. Another important pathway for the fusion of autophagosomes and lysosomes is syntaxin 17 through its interaction with the lysosomal synaptosomal-associated protein 29 (SNAP29) and SNARE vesicle-associated membrane protein 8 (SNARE VAMP8) [14-17]. Inside the autolysosome, there is an acidic environment and hydrolases that originate from the lysosome. These cause the degradation of the inner membrane of the autophagosome and the engulfed cytoplasmic organelles. The degraded products are then expelled from the autolysosome through lysosomal permeases back into the cell’s cytoplasm. The expelled products are used for energy production or biosynthetic processes in the cells. For simplicity, the macro-autophagy process will be referred to as autophagy from here onward [18].

Autophagy can function both selectively and nonselectively. During periods of nutrient deprivation, nonselective autophagy targets any organelles in the cells to produce energy. Selective autophagy targets specific damaged organelles, recycling them to produce optimal cellular function. One such example is mitophagy. Mitophagy involves the clearance of damaged mitochondria. Mitochondrial function is known to be associated with the life span of species [20-23]. Indeed, there is a piece of ample evidence to indicate that the optimal function of autophagy maintains homeostasis in eukaryotic cells (including cardiovascular cells), and this correlates with an increased life span [24].

The discussed knowledge in conjuncture with evidence suggests that reduced autophagy correlates with increased cardiovascular pathologies upon aging, which warrants investigations into autophagy as a regulator of cardiovascular diseases [25].

Implications of aging on the cardiovascular system

Aging-Associated Altered Cardiac Changes

As mentioned, increased age enhances the risk of developing cardiovascular disease. Specifically, the incidence of cardiovascular disease is 10 per 1000 men and four per 1000 women at the age of 45-54 years, with this increasing to 74 men and 65 women per 1000, respectively, at the age of 85-94 years [26]. This is because as humans age, structural and functional alterations occur in the heart and vasculature that predispose individuals to an increased risk of cardiac and vascular diseases, including strokes, atrial fibrillation, coronary artery disease, and atherosclerosis [27]. The structural changes include increased size and altered shape of the heart with age. This is due to the increased cardiomyocyte size with a disproportionate increase in the interventricular septum concerning the wall of the heart, resulting in undesired cardiac stress and contractile dysfunction [28]. Other structural changes of the heart associated with aging include left ventricular wall thickening accompanied by increased collagen deposition and fibrosis [27,28]. Even with such grave structural changes, the cardiac systolic function remains unchanged at rest as people age, as demonstrated by studies that showed the stability of cardiac ejection fraction at rest [27,28]. However, during periods of increased oxygen demand, the cardiac output of the aging heart decreases because of impaired maximal heart rate.

In contrast to systolic function, cardiac diastole function is affected by age. As people age, their heart fills more slowly in the diastolic phase. Cardiac diastole happens in two phases: (1) the first phase is called the “passive phase” when blood falls from the atria into the ventricles, and the second phase is called the “active phase” where the atria push the blood into the ventricles. With aging, the first phase of diastole is reduced, resulting in an increased need for the second phase to compensate for the change. As the atria contract more to fill the ventricles in the second stage of diastole, there is subsequently atrial remodeling and enlargement [29,30]. Other age-associated factors can also affect cardiac functions, such as fat accumulation at the sinoatrial node, calcification of the left side of the heart, and loss of pacemaker cells. These changes can lead to conduction problems in the hearts of aging individuals resulting in an overall increased risk of atrial arrythmias/fibrillations, ventricular arrhythmia, and paroxysmal supraventricular tachycardia in the aging population [31].

Furthermore, with aging, both cardiac systole and diastole are prolonged, which is because the action potential, the transient increase in cytosolic calcium, and the contraction rate of the heart are all prolonged [32,33]. In addition, with age, the effects of the sympathetic nervous system on the heart diminish. This is considered to be due to the aging-associated reduced response of postsynaptic β-adrenergic receptors responsible for left ventricular wall contractility, heart rate increase, and arterial afterload decrease during periods of increased cardiac demand (i.e., exercise) [34-35].

**Figure 3:**
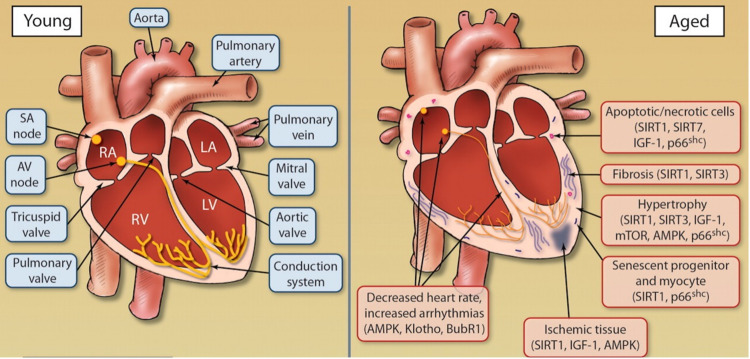
Changes that occur in the heart with aging SIRT, Sirtuin; SA node, sinoatrial node; AV node, atrioventricular node; IGF, insulin-like growth factor; MTOR, molecular target of rapamycin; AMPK, AMP-activated protein kinase. Source: This figure was taken from North et al. 2012 [30].

Collectively, the mentioned aging-associated cardiac changes culminate to produce decreased cardiac output and subsequently reduced peak oxygen consumption during periods of high demand. As a result, older people experience an inability to fully function in their daily lives and carry out tasks that they would previously do unaffected as enumerated in Figure 3.

Aging-Associated Altered Vascular Changes

In addition to aging-associated cardiac changes, the vascular structure is also affected by aging. These changes, coupled with cardiac changes, impede the normal physiological function of the cardiovascular system and, hence, increase the risk of many pathologies, including atherosclerosis, ischemic heart disease, hypertension, and heart failure [29].

A key vascular change with age is an increase in the arterial wall thickness and dilatation of the lumen [34]. Epidemiological studies indicate that the intimal medial thickness of the arteries increases two to three times from 20 to 90 years of age. In aged individuals, this increase in intimal thickness and dilatation of the lumen is correlated with the development of the early stages of atherosclerosis. In addition, increased arterial stiffness and reduced vascular distensibility are associated with aging. These changes are due to age-associated structural alterations in the vascular media such as increased collagen deposition and reduced elastin. Calcification of the media is also an important feature of vascular aging. Such changes lead to a vicious cycle, whereby arterial stiffening results in a widening of the pulse pressure because of increased systolic pressure and decreased diastolic pressure. This sequence of events further enhances intimal medial thickening that also widens pulse pressure. This vicious cycle is linked to the prediction of altered cardiovascular events later in life [29,36,37].

**Figure 4:**
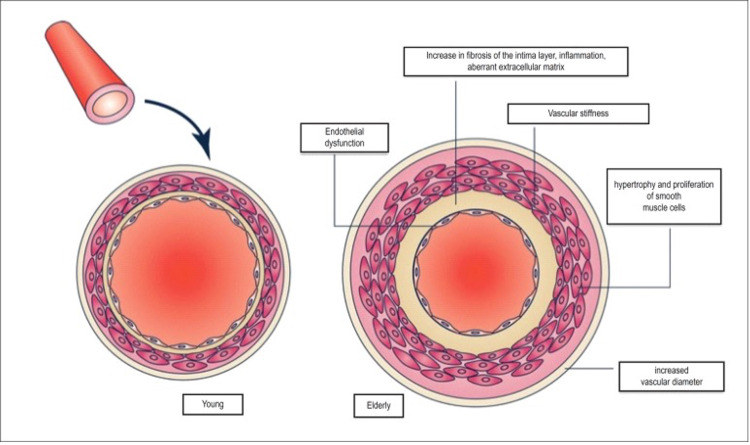
Changes that occur in the vascular system with aging Source: This figure was taken from Mikael et al. 2017 [40].

Another key element of vascular aging is endothelial cell (EC) dysfunction. The hallmark of this is the diminished production of nitric oxide by ECs. Nitric oxide is responsible for vascular dilatation, tone regulation, inhibiting vascular inflammation, thrombotic events, and abnormal cell proliferation. All of these regulatory and protective features are lost with aging. Nitric oxide is also responsible for diminishing reactive oxygen species (ROS) in ECs. As nitric oxide production decreases with aging in ECs, the generation of ROS increases. ROS are then responsible for the oxidation of low-density lipoproteins and promotion of adhesion molecules such as intracellular adhesion molecule-1 and vascular EC adhesion molecule-1. These changes are associated with early atherosclerosis in aging individuals [38]. Finally, ECs demonstrate senescence with aging and hence lead to reduced proliferation and barrier disruption. This allows the migration of subendothelial smooth muscle cells, resulting in extracellular matrix deposition and further intimal thickening [39]. Importantly, as people age, the decrease in arterial elasticity (Ea) is matched to the decrease in left ventricular elasticity (Elv), maintaining the Ea/Elv ratio, allowing the cardiovascular system to function as best as possible. This is of particular significance in the context of arterial and ventricular coupling. However, when aged individuals are stress-challenged, this coupling is lost, which partly explains the cardiovascular compromise during periods of increased oxygen demands among elderly individuals [28]. Parity has been drawn between heart vascularity in young and elderly as shown in Figure 4.

Autophagy: a protective molecular pathway in cardiovascular health

To understand how aging-associated cardiovascular disease can be prevented through targeted autophagy at the molecular level, it is essential to understand the correlation between the evolutionary conserved molecular pathways associated with cardiovascular health during aging and how they function.

Molecular Target of Rapamycin (MTOR)

**Figure 5:**
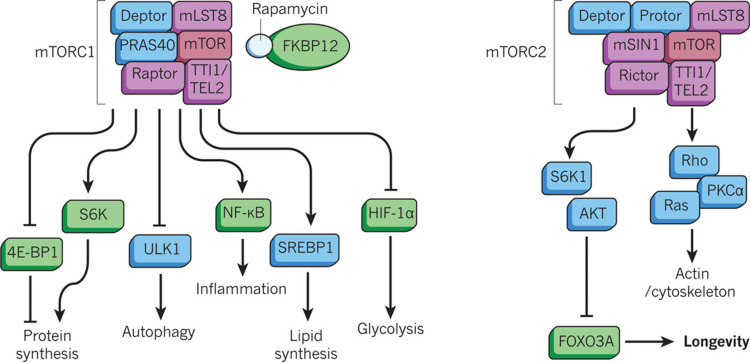
The MTOR complex MTOR, Molecular target of rapamycin. Source: This figure was taken from Johnson et al. 2013 [41].

MTOR is a serine/threonine kinase of the phosphatidylinositol-3-OH kinase-related family. MTOR is expressed via the genes target of rapamycin 1 (TOR1) and target of rapamycin 2 (TOR2). Two complexes of MTOR exist, MTOR1 and MTOR2. This review will discuss the MTOR1 complex. As mentioned in the introduction, rapamycin (an immunosuppressant) inhibits the MTOR1 complex activity. This inhibition is brought about through the binding of rapamycin with the FK-506-binding protein (FKBP12), which interacts with MTOR1 complex, an interaction that inhibits its activity. Another endogenous inhibitor of the MTOR1 complex is the AMP-activated protein kinase. Activators of the MTOR1 complex include insulin and growth factors [41]. The MTOR1 complex and its associated pathways are involved in a plethora of cellular functions, including growth, proliferation, motility, survival, and autophagy, all of which are illustrated in Figure 5.

These responses are all associated with the promotion of normal cellular function (including normal cardiovascular function) [27]. Numerous studies have demonstrated the relationship between the MTOR complex and life span in different species. For example, the life span of the nematode *Caenorhabditis elegans* is prolonged after deletion of the MTOR (let-363) or the MTORC1 component raptor from the MTOR complexes [42]. As compared with mice expressing the normal MTOR gene, mice that were genetically engineered to show reduced transcription of MTOR genes had a prolonged life span [43]. Caloric restriction or administration of rapamycin has been shown to downregulate the MTOR-complex pathway and promote cardiovascular health with aging [27]. In addition, rapamycin inhibition of the MTOR1 complex has protective effects on the vascular system [44]. It is clear that the MTOR pathway plays an important role in longevity and cardiovascular health with aging.

Sirtuins

**Figure 6:**
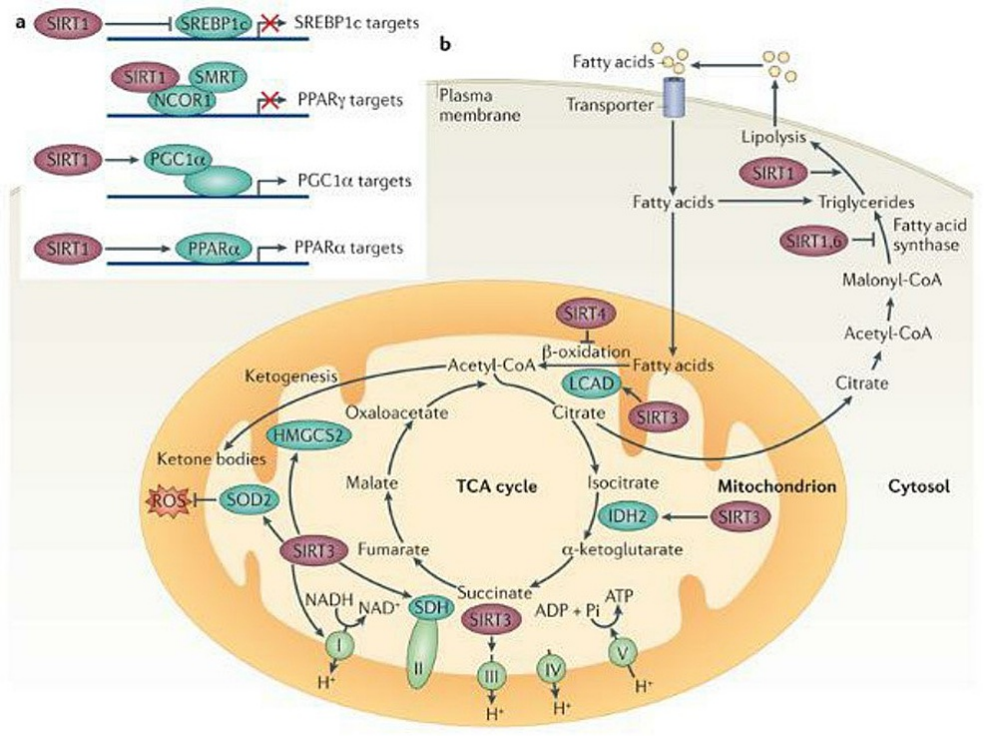
Actions of the sirtuins TCS, Tricarboxylic acid; SIRT, sirtuin; SREBP1, sterol regulatory element-binding protein 1; PPAR-γ, peroxisome proliferator-activated receptor gamma; PGC1α, peroxisome-proliferator-activated receptor-γ coactivator-1 alpha; PPARα, peroxisome proliferator-activated receptor alpha; IDH, isocitrate dehydrogenase; HMGCS2, 3-hydroxy-3-methylglutaryl-CoA synthase 2; LCAD, long-chain acyl-CoA dehydrogenase; SDH, succinate dehydrogenase; ROS, reactive oxygen species. Source: This figure was taken from Houtkooper et al. 2012 [45].

Sirtuins are a family of proteins that include sirtuin 1 to sirtuin 7. Sirtuins are found in different compartments of cells including the nucleus, cytoplasm, and mitochondria. Their role is to control and regulate the metabolism of fat and glucose according to the energy levels in the cells. They also play a role in cell aging. Sirtuins exert their effects on cells through control of transcription factors, deacetylation of histones on DNA, and regulation of cytoplasmic proteins [45]. Of the seven sirtuins identified, the most studied is sirtuin 1. Sirtuin 1 deacetylates and deactivates the p53 tumor-suppressor gene during periods of cellular oxidative stress and DNA damage. This impairs the apoptotic function of cells and aids in tumor-protective properties [46]. Figure 6 enumerated the following: Sirtuin 1 also controls and activates through deacetylation peroxisome proliferator-activated receptor-gamma coactivator (PGC1a). PGC1a is a transcriptional factor that promotes gene expression in the mitochondria, thereby increasing mitochondrial biogenesis and subsequently improving their function. This function of sirtuin 1 is also important because, as discussed previously, the optimal function of the mitochondria is correlated with an enhanced life span [47]. Sirtuin 3 is another sirtuin that plays an integral role in mitochondrial function. Sirtuin 3 deacetylates a mitochondrial protein called long-chain acetyl-coenzyme A dehydrogenase, the deacetylation of which allows for the use of fatty acids during periods of cellular energy depletion. Furthermore, sirtuin 3 is involved in the production of ketone bodies through deacetylation of the enzyme 3-hydroxy-3-methylglutaryl CoA synthase 2. During periods of starvation, ketone bodies are an important energy source, especially for brain survival. Finally, sirtuin 3 protects cells from damage due to oxidative stress in periods of starvation by activating the antioxidant superoxide dismutase 2 [45].

Alongside the beneficial effects of sirtuins at the cellular level, supplementation of nicotinamide riboside (an activator of sirtuins) can prolong the life span in mice [48]. The literature also shows that in addition to promoting increased life span, sirtuins can promote increased health quality [49]. In relation to the cardiac system, a moderate increase in the expression of sirtuin 1 in mice prevents aging of the heart [50]. Along the same line of thought, deactivation of sirtuin 3 and sirtuin 6 results in cardiac hypertrophy, fibrosis, and reduced life span [27]. With regard to the vascular system, the expression of sirtuin 1 decreases with aging. This is associated with vasodilatory dysfunction and promotion of a vascular EC senescence-like phenotype [27]. The above studies demonstrate the integral role of sirtuins in the cardiovascular system and aging.

Insulin-Like Growth Factor-1 Signaling

**Figure 7:**
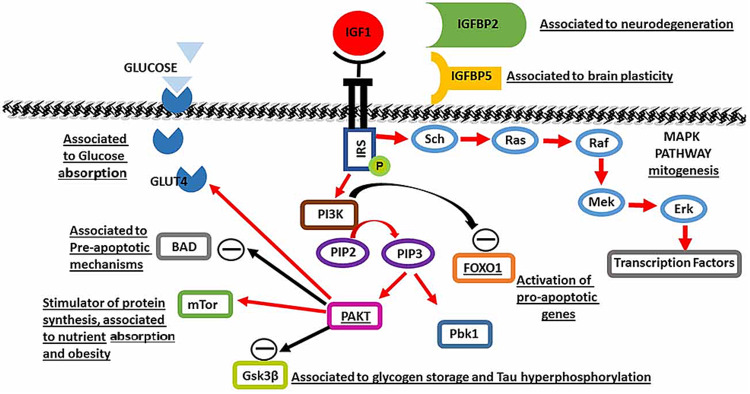
Insulin-like growth factor-1 intracellular signaling pathway MTOR, Molecular target of rapamycin; IGFBP, insulin-like growth factor binding proteins; IRS, insulin receptor substrate. Source: This figure was taken from Wrigley et al. 2017 [53].

In mammals, the growth hormone/insulin-like growth factor-1 (GH/IGF-1) signaling pathway is an evolutionarily conserved pathway associated with cellular metabolism regulation. Manipulation of this GH/IGF-1 pathway can promote the extension of the life span [51]. Growth hormone production by the anterior pituitary stimulates the production of IGF-1 by the liver and tissues in the body. After production, IGF-1 binds and activates IGF-1 tyrosine kinase receptors on cellular membranes. This leads to an intracellular cascade of events accompanied by the upregulation of intracellular substrates via phosphorylation. Figure 7 denotes the process in which the phosphorylated substrates lead to the activation of two pathways, the phosphatidylinositol 3-kinase-protein kinase B (PI-3K-PKB) AKT pathway and the RAS-mitogen-activated protein kinase (RAS-MAPK) pathway. The PI-3K-PKB AKT pathway is associated with metabolic effects, whereas the RAS-MAPK pathway is associated with mitogenic effects. However, because there are genetic variations in components of the IGF-1 signaling pathway, the functions and potential manipulations of this pathway for the promotion of longevity are complex [52].

The beneficial effects of the GH/IGF-1 pathway on life span are achieved through its suppression. For example, there is evidence that the genetic suppression of IGF-1 in mice is correlated with longevity [54]. In addition, low IGF-1 levels in humans predict an increased life span, showing that the beneficial effects of suppressing the IGF-1 pathway are reproducible in humans as well [55]. Regarding the heart, overexpression of the IGF-1 pathway induces cardiac hypertrophy and improved cardiac output in young transgenic mice. However, as the mice aged, this beneficial effect diminished, and deleterious cardiac pathologies emerged. This includes decreased systolic performance and increased fibrosis of the heart. Even if short-term IGF-1 overexpression is beneficial for the heart, the effects are disastrous in the long term [56]. IGF-1 is also associated with benefits for the vascular system. Mice lacking the IGF-1 receptor on ECs showed increased production of nitric oxide, a phenomenon that, as discussed previously, benefits the vasculature through antioxidant activities and increased EC health. This beneficial effect was also observed in the human vasculature by using siRNA to inhibit IGF-1 receptor activity [57]. However, further studies are needed to investigate the role of IGF-1 on the cardiovascular system to further assess how the beneficial effects of IGF-1 can be extracted.

AMP-Activated Protein Kinase (AMPK)

**Figure 8:**
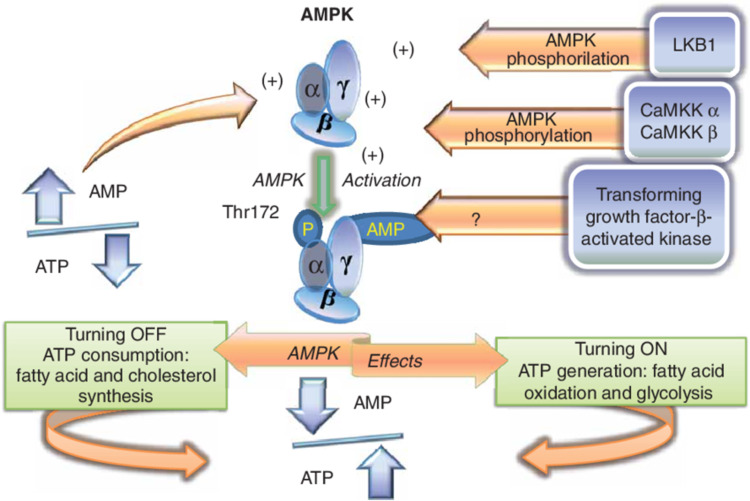
AMP-activated protein kinase function AMP, Adenosine monophosphate; AMPK, AMP-activated protein kinase. Source: This figure was taken from Antonioli et al. 2015 [59].

The AMPK is another intracellular molecule that aids in cell survival and function. This kinase is responsible for controlling the energy sources of the cells during periods of energy scarcity, in that it is activated when cells are low on their energy sources. Specifically, regulation of AMPK is achieved through the AMP-ATP gradient. When AMP is high and ATP is low, AMPK is activated, and vice versa. Figure 8 shows that once the AMPK is activated, it phosphorylates and thus upregulates the enzymatic function of hydroxymethylglutaryl-CoA reductase and acetyl-CoA carboxylase. These enzymes are then responsible for the synthesis of sterol and fatty acids in cells. Sterol and fatty acids are subsequently used for cellular energy production, shifting the cells’ energy source from traditional sources in times of low energy availability and thus promoting cell survival [58].

In mice, metformin, which activates the AMPK, prolongs both health and life span. However, to extract the beneficial effects, a moderate dose of metformin should be used, as higher doses are associated with adverse effects, such as nephrotoxicity. This shows that although increased AMPK activation is associated with life span and longevity, accomplishing this effect should be closely considered as to not cause damage elsewhere [60]. Caloric restriction is another method of inducing AMPK and subsequent increase in life span [27]. The AMPK pathway is also associated with the health of the cardiovascular system. Here, decreased AMPK activity causes cardiomyocyte hypertrophy, contractile dysfunction, mitochondrial damage, and intracellular calcium mishandling [61]. In addition, the administration of metformin to aged mice demonstrates improved cardiac contractility and reduced cardiac fibrosis [27]. In the vascular system, the induction of AMPK by aminoldazole carboxamide ribonucleotide and curcumin exerts protective effects in both mice and humans. These include improved vasodilation, enhanced nitric oxide production, and reduction of oxidative stress [27]. Based on the above, it can be concluded that induction of AMPK plays a key role in regulating cardiovascular health and longevity.

Autophagy and aging

As discussed, autophagy is reduced with aging. Therefore, it is important to understand how this aging-associated reduced autophagy affects the cardiovascular system. The molecular pathways discussed previously that facilitate increased life span and cardiovascular health are associated with autophagy function. As people age, the beneficial effects of these pathways are reduced and thus can explain in part why autophagy is reduced as well. For example, with aging, the MTOR-1 complex is significantly upregulated, and the AMPK pathway is downregulated, thus reducing the beneficial effects it has on longevity and the cardiovascular system. This effect is brought about because both the MTOR-1 complex and the AMPK pathway control autophagy through the phosphorylation of UNC-51, such as autophagy-activating kinase 1 complex. In aged individuals, this complex remains inactivated, and thus autophagy is reduced [27].

Several transcription factors have been identified that promote enhanced gene expression and subsequent upregulation of autophagy. These transcription factors are suppressed as people age and therefore reduce autophagy [62]. One such transcription factor is the transcription factor EB (TFEB). TFEB belongs to the microphthalmia-associated transcription factor family. MTOR kinase is responsible for the regulation of TFEB. During nutrient-rich conditions, MTOR kinase phosphorylates TFEB in the cytoplasm of cells and prevents its activation. Conversely, when cells are nutrient-starved, the phosphorylation of TFEB is prevented. This results in the translocation of TFEB to the nucleus, where it enhances the expression of several genes that play a role in the process of autophagy. Specifically, TFEB is responsible for promoting autophagy genes that are involved in lysosome formation, autophagosome biogenesis, identification of cytoplasmic organelles by the autophagosome, fusion of autophagosome with lysosomes, and ejection of the degraded products from autolysosomes back into the cytoplasm [62]. TFE 3, another member of the microphthalmia-associated transcription factor family, with similar functions to TFEB, shows reduced activity with aging. Another important family of transcription factors that play a pivotal role in activating genes for autophagy is the forehead transcription factors family (FOXO). The forkhead transcription factors are directly activated by AMPK, and they are inhibited by AKT-mediated phosphorylation and lysin acetylation, which occurs due to decreased nicotinamide adenine dinucleotide (NAD) and consequent inactivation of SIRT 1 [62,63]. With aging, the promoters of the FOXO family reduce their activity, and the inhibitors increase their activity [27]. FOXO 3, a member of the FOXO family, improves the lysosomal function in autophagy. This is achieved through the improved lysosomal ability to break down the engulfed organelles and proteins. Other members of the FOXO family, FOXO 1, FOXO 4, and FOXO 6, play important roles in cytoplasmic protein homeostasis, which subsequently affects improved cell function [64].

With aging, there is an accumulation of cellular damage that impedes normal cellular function, which negatively affects autophagy. One such example is ROS (superoxide and hydrogen peroxide). As people age, the accumulation of ROS increases. This increase is mainly induced by oxidative phosphorylation to produce ATP in the mitochondria. The increased accumulation of ROS causes DNA damage, misfolding of proteins, and damage to the mitochondrial respiratory chain. The result is a vicious cycle whereby organelles (mitochondria in general) cannot function properly and produce an even greater amount of ROS. To cope with the increased need to recycle dysfunctional proteins and organelles, an increased autophagic function is required. Over time, increased autophagic expression is exhausted and thus subsequently decreases with aging [63,65]. An alternative explanation is that the accumulation of ROS impedes mitophagy function. This is the result of damage to critical proteins (e.g., mitofusin 1, dynamin-related protein 1, and fission 1) responsible for mitophagy regulation [27]. As people age, the lysosomal function is also impeded. When lysosomes engulf damaged mitochondria for degradation, the increased misfolded proteins and peroxides from the engulfed mitochondria crosslink, leading to the formation of lipofuscin. The buildup of lipofuscin in lysosomes impedes their function in autophagy. In addition, with aging, downregulation of the lysosome-associated membrane protein 2a is observed, which results in additional impairment in autophagy [66].

Intracellular calcium signaling plays an important role in the regulation of autophagy. Calcium is stored mainly in the endoplasmic reticulum in the cytoplasm. The release of calcium from the endoplasmic reticulum is mediated by the activity of inositol 1, 4, 5-triphosphate receptors. The production of inositol 1, 4, 5-triphosphate at the cell’s plasma membrane stimulates inositol 1, 4, 5-triphosphate receptors, which in turn induces the release of intracellular calcium. This activation of the inositol 1, 4, 5-triphosphate receptors results in the inhibition of autophagy. The calcium-mediated inhibition of autophagy is suggested to be caused by either of two mechanisms. First, increased calcium signaling in cells is suggested to increase ATP production, which inactivates AMPK and, therefore, inhibits autophagy. Second, it is suggested that calcium release from the endoplasmic reticulum results in the formation of the anti-autophagic complex BCL2-BECN1 [67]. With aging, the activity of inositol 1, 4, 5-triphosphate receptors increases in mice and humans, indicating an aging-associated relationship between calcium and decreased autophagy [27]. Epigenetic changes also play an important role in the function of autophagy with aging. As people age, there is increased production of nucleo-cytosolic acetyl coenzyme A, which in turn acetylases DNA histones of the ATG7 autophagy gene. This results in decreased ATG7 gene expression and subsequently decreased autophagy with aging [27,62,68].

The above studies demonstrate that autophagy decreases in organisms (including humans) with aging. To understand the importance of this, we will discuss the impact of reduced autophagy on the cardiovascular system. One such study looked at the effects of reduced autophagy on the cardiac system in mice. In this study, scientists compared the effects on the cardiac system between ATG5 knockout mice and normal control mice [66]. ATG5 is an essential component of autophagy that plays a crucial role in associating with ATG12 to promote the fusing and formation of autophagosomes by phagophores. Mice lacking ATG5 would therefore have reduced the autophagic function. Here, we will use the term reduced, and not completely blocked (autophagy), because recycling of the damaged organelles and proteins in the cell’s cytoplasm could still occur as a result of mitochondrial fusion and fission, chaperon-mediated autophagy, and ATG5-independent autophagy. The study evaluated the condition of mouse hearts at three and 10 months of age [66]. At three months of age, even though the hearts of the ATG5-deficient mice did not show any functional or structural changes, the molecular functions in the heart cells were compromised when compared with controls. Specifically, the mitochondria in the heart cells of ATG5-deficient mice were malfunctioning with the respiratory chain (including complexes I+III and II+III) of mitochondria that were not working properly. Heme oxygenase-1, an enzyme responsible for decreasing oxygen consumption and producing ROS, was also elevated in the ATG5-deficient mice; consequently, increased oxidative stress was observed. As a result, cardiomyocyte apoptosis was noted in the hearts of ATG5 knockout mice at three months of age. At 10 months of age, ATG5 knockout mice also began to develop deleterious structural and functional cardiac changes (in contrast to the controls), exhibiting increased molecular markers (atrial natriuretic factor, brain natriuretic peptide, and skeletal α-actin) associated with heart remodeling. When compared with controls, ATG5-deficient mice developed heavier hearts, atria and ventricular dilatation, increased end-diastolic and end-systolic left ventricular size, and diminished heart ejection fractions. The heart contractility of ATG5-deficient mice was also compromised because of the increased cross-sectional area of cardiomyocytes, fibrosis, and collagen deposition. These changes in the hearts of ATG5-deficient mice resulted in heart failure and subsequent premature death when compared with controls [69]. Other studies have also provided evidence supporting the correlation between decreased autophagy and enhanced cardiac disease [70-73].

**Figure 9:**
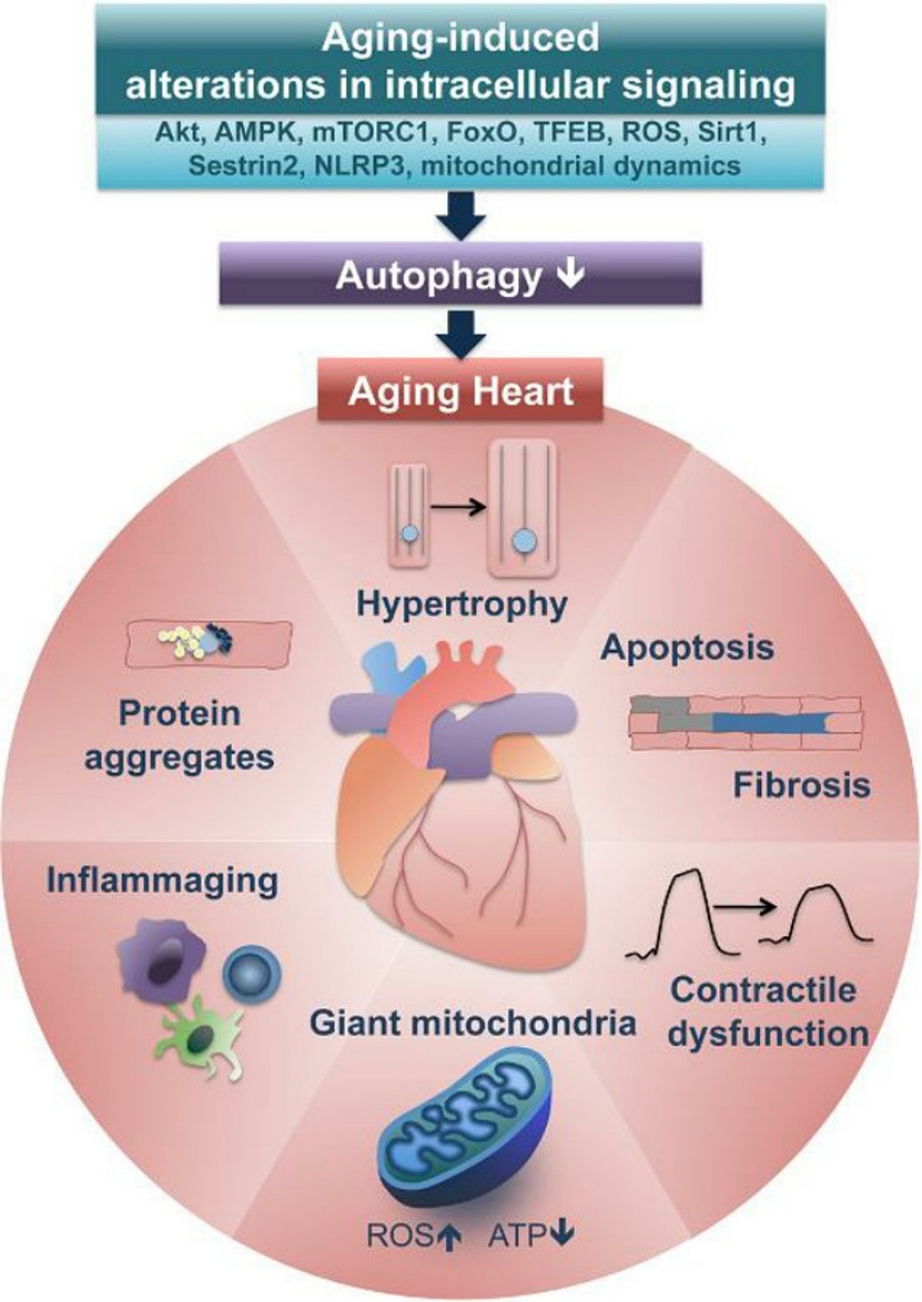
Implications of reduced autophagy on the heart caused by aging MTOR, Molecular target of rapamycin; AMPK, AMP-activated protein kinase; TFEB, transcription factor EB, Sirt1, Sirtuin 1; ROS, reactive oxygen species. Source: This figure was taken from Miyamoto (2019) [78].

Reduced autophagy also appears to have a negative effect on the vascular system. In a study involving ATG7 knockout mice (ATG7 deletion has similar autophagy-reducing effects as ATG5), dysfunctional vascular contractility was noted under conditions of ATG7 deficiency. These effects were the result of increased inositol 1, 4, 5-triphosphate signaling for cytoplasmic calcium release, which altered the contractility of smooth muscles in the vascular system [74]. In another study, PRKAA (protein kinase, AMP-activated, a catalytic subunit) gene was removed from the DNA of mice, so they did not code for it. PRKAA is responsible for regulating the recycling of damaged mitochondria through autophagy. The PRKAA-deficient mice experienced an increased accumulation of dysfunctional mitochondria in vascular ECs. As discussed previously, this resulted in the accumulation of ROS, which subsequently hindered the production of nitric oxide [75]. A reduction in nitric oxide affects the vascular system in many ways (as discussed previously), which culminates in the manifestation of early atherosclerosis [38]. Overall, decreased autophagy is related to vascular disease. Another study on genetically modified mice that exhibited reduced autophagy showed that these mice also exhibited an increased endothelial-mesenchymal transition. The endothelial-mesenchymal transition is a process in which ECs change their phenotype to different kinds of cells (e.g., myofibroblasts and smooth muscle cells). Such a phenomenon results in many vascular problems, including loss of EC adhesion, leaking capillaries, and vascular malformations, events that are linked to atherosclerosis [76,77] as summarized by Figure 9.

Beneficial effects of stimulating autophagy

The above sections have discussed the deleterious effects of aging on both the structural and functional aspects of the cardiovascular system. In addition, this review has provided examples of numerous works that have demonstrated the negative effects of reduced autophagy on the cardiovascular system. The following sections will discuss the different ways in which autophagy can be induced as a therapeutic and preventative strategy for reducing aging-associated cardiovascular disease.

Caloric Restriction

Reduction in calorie intake is one way, in which autophagy can be induced in organisms (and humans). Caloric restriction is defined as a 30%-50% reduction in the calories an organism would require daily, without causing malnutrition [79]. Caloric restriction activates autophagy through the depletion of energy available to the cells. This causes an increase in NAD, which subsequently activates sirtuin-1 and its associated autophagic pathway [80]. Increased autophagy can exert beneficial effects on the heart and protect it from age-related cardiac deterioration; as such, caloric restriction can decrease the aging-associated cardiac diastolic dysfunction [79]. In addition, in a study observing the effects of caloric restriction on rodents, the animals displayed decreased cardiac fibrosis, cardiac hypertrophy, contractile dysfunction, and cardiomyopathy with aging [81]. Similarly, there is a piece of significant evidence for the beneficial effects of caloric restriction on the detrimental effects of aging on the vascular system. For example, a lifelong caloric restriction of 40% in rats showed preserved production of endothelial nitric oxide in the vascular system, thus preserving vascular vasodilation and endothelial function. Moreover, caloric restriction showed a protective effect on age-dependent systolic and diastolic arterial pressure, vascular stiffening, and vascular wall hypertrophy [82]. The protective effects that autophagy exerts on the functional and structural elements of the cardiac and vascular system indicate that it would be a valuable preventative target against cardiovascular diseases associated with aging.

Intermittent Fasting

Daily caloric restriction is not the only way autophagy can be induced in individuals. Caloric intake can be manipulated in different ways to achieve autophagy induction. Intermittent fasting is an alternative means through which organisms can reduce their total weekly calories rather than daily calories. An example of that is alternate-day fasting, in which individuals interchange between eating normally for one day and then fasting the next day [83]. Alternate-day fasting promotes autophagy through reduced phosphoinositide-3-kinase signaling [27]. Intermittent fasting has similar protective effects on the cardiovascular system as calorie restriction does. Studies on rodents and mice indicate that intermittent fasting can protect the heart during myocardial infarction. Specifically, rats placed on alternate-day fasting three months before myocardial infarction exhibited reduced myocardial infarct size and reduced cardiac cell death when compared with controls. In addition, after myocardial infarction, the rats on the alternate-day fasting regime showed no progression of myocardial infarct size and no cardiac remodeling, thus exhibiting better cardiac function than the controls did [84]. The beneficial effects of alternate-day fasting can also be harvested when this regime is implemented in rats two weeks after myocardial infarction, with the rats showing improved cardiac function and survival against the control group [84]. Similarly, in humans, intermittent fasting hinders age-related degradation of the heart by reducing inflammation, ROS, and fibrosis [84]. Homocysteine, interleukin-6, and C-reactive proteins are proinflammatory cytokines that contribute to the development of atherosclerosis in the human vasculature. In a study on human subjects, the intermittent fasting (increased autophagy) group exhibited reduced levels of these proinflammatory cytokines when compared with controls, indicating protective effects of intermittent fasting on the vascular system [85]. Hence, in addition to caloric restriction, intermittent fasting also protects hosts from age-associated degradation of the cardiovascular system through increased autophagy induction.

Pharmacologic activation of autophagy

Although enumerated caloric restriction and intermittent fasting have shown promising effects on inducing increased rates of autophagy with subsequent cardiovascular protection against aging, such strategies are not easily implemented in large numbers of people for the prevention of cardiovascular disease. To solve this problem, pharmacologic agents have been identified that induce autophagy and harness its beneficial effects on the cardiovascular system in a similar manner to caloric restriction and intermittent fasting. Key examples of such pharmacologic agents are as follows.

Spermidine

Spermidine upregulates various transcription factors associated with autophagy through the inhibition of histone acetyltransferases, which in turn cause deacetylation of histone H3 [86]. Supplementation of spermidine on mice showed improved diastolic function, reduced myocardial hypertrophy, and reduced left ventricular passive stiffness. Moreover, old mice supplemented with spermidine had values of ventricular arterial coupling similar to those of young mice. This is extremely important, as the ventricular arterial coupling is a prognostic factor for heart failure. In addition, spermidine prevented age-related degradation of cardiomyocytes through a series of mechanisms. First, when compared with controls, spermidine supplementation in mice showed improved respiratory function through increased respiratory complex I in cardiac cell mitochondria. Second, spermidine-supplemented mice showed decreased levels of the proinflammatory cytokine tumor necrosis factor-α. Third, spermidine-supplemented mice showed improved levels of cardiomyocyte elasticity by exhibiting a conserved cytoskeletal apparatus (myosin heavy chain proteins, ankyrins, integrins, and dystonin) with aging. Finally, spermidine supplementation reduces blood pressure in mice, which delays the manifestation of heart failure [87]. In relation to the vascular system, spermidine supplementation showed preserved vascular EC function with aging, reversed vascular stiffening and collagen deposition with aging, and decreased oxidative stress. The beneficial effects of spermidine on the vascular system are induced by maintained nitric oxide production with aging [88].

Rapamycin

As discussed previously, rapamycin inhibits the MTOR-ULK1 pathway and subsequently induces autophagy in humans and other organisms [41]. Rapamycin-supplemented mice showed a reduced accumulation of inflammatory markers associated with aging in the heart. Second, mice administered with rapamycin late in life had reduced left ventricular wall contractility, improved diastolic ventricular blood filling, and improved cardiac ejection fractions than controls did. The beneficial effects of rapamycin supplementation on cardiac function are a consequence of the upregulation of the RAD protein in cardiomyocytes. The RAD protein is responsible for inhibiting hypertrophy and improving cardiomyocyte function in the heart. It exerts its effects through the control of calcium channels, tropomyosin, and calmodulin signaling in the heart cells [89].

Resveratrol and Nicotinamide Mononucleotide

**Figure 10:**
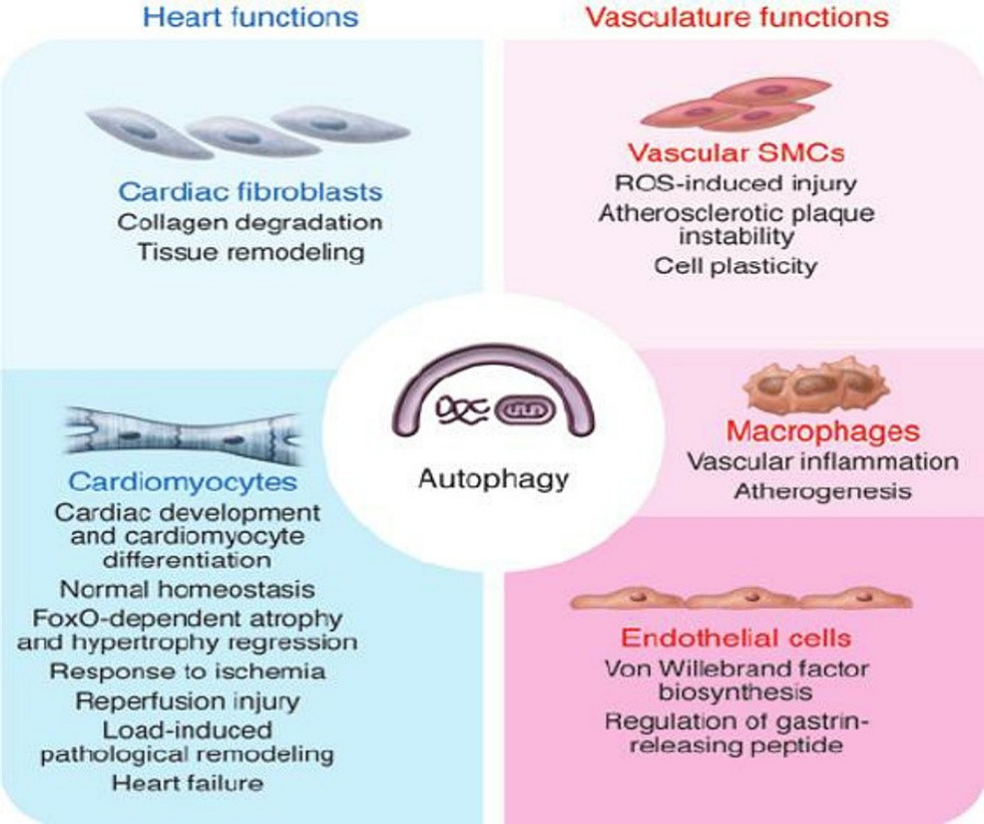
Action of autophagy on the cardiovascular system SMC, Smooth muscle cells; ROS, reactive oxygen species. Source: This figure was taken from Lavandero et al. 2015 [93].

Resveratrol and nicotinamide mononucleotide agents induce autophagy in humans through activation of the sirtuin-1 pathway as discussed [27]. During aging, resveratrol mediates cardiac cell health and prevents cardiac functional and structural decline [90]. In addition, resveratrol shows similar preservation characteristics as intermittent fasting on cardiac function after myocardial infarction. Resveratrol also protects the vascular system from the formation of atherosclerotic plaque by reducing inflammation, reducing vascular EC dysfunction, reducing vascular smooth muscle cell proliferation, and reducing platelet aggregation [91]. Nicotinamide mononucleotide supplementation reduces collagen type 1 and increases deposition of elastin in the aorta of animals to levels exhibited by young healthy animals, thus reducing the age-associated aortic stiffness. Second, nicotinamide mononucleotide reduces oxidative stress and increases the production of nitric oxide in vascular ECs, which subsequently preserves the endothelial vascular cell function with aging [92]. The summarized action of autophagy is depicted in Figure 10.

Together, it is clear that promoting autophagy through caloric restriction, intermittent fasting, or pharmacologic agents reverses the reduction of autophagy that naturally happens with aging and subsequently protects the cardiovascular system.

## Conclusions

We know that the world’s population is currently living longer. This is especially problematic, given the increase in the prevalence of chronic conditions resulting from an increased aging population, thus negatively affecting the healthspan and quality of life of the affected individuals. This review discussed the effects of aging on the cardiovascular system and established an increased predisposition of cardiovascular pathologies in the geriatric population due to molecular, structural, and functional changes in both the cardiac and vascular systems. Longevity molecular pathways exist to maintain the homeostasis of the cardiovascular system and promote health. Autophagy is at the interlink of these pathways. Although autophagy is downregulated as people age, stimulation of this pathway through caloric restriction, intermittent fasting, and supplementation of pharmacologic agents can reinstate autophagy in older individuals. Induced autophagy promotes the longevity of cardiovascular health, thereby instigating the role of autophagy in the prevention of chronic conditions such as cardiovascular ailments. Strong evidence for this notion has emerged from studies using genetically modified mice defective in genes, transcription factors, and proteins essential for autophagy and as such failed to show improvements in cardiovascular health when caloric restriction, intermittent fasting, and pharmacologic agents are implemented.

To summarize, future research should be directed toward human studies or human tissues in vitro. This will allow for a clearer understanding of the role of autophagy on the longevity pathways and cardiovascular disease prevention in humans. In addition, future research should evaluate how the beneficial effects of autophagy can be implemented reproducibly and on large scales in the population.
